# Platelet-derived growth factor receptor-β and epidermal growth factor receptor in pulmonary vasculature of systemic sclerosis-associated pulmonary arterial hypertension versus idiopathic pulmonary arterial hypertension and pulmonary veno-occlusive disease: a case-control study

**DOI:** 10.1186/ar3315

**Published:** 2011-04-14

**Authors:** Maria J Overbeek, Anco Boonstra, Alexandre E Voskuyl, Madelon C Vonk, Anton Vonk-Noordegraaf, Maria PA van Berkel, Wolter J Mooi, Ben AC Dijkmans, Laurens S Hondema, Egbert F Smit, Katrien Grünberg

**Affiliations:** 1 Department of Pulmonary Diseases, VU University Medical Center, De Boelelaan 1117, 1081 HV, Amsterdam, The Netherlands; 2 Department of Rheumatology, VU University Medical Center, De Boelelaan 1117, 1081 HV, Amsterdam, The Netherlands; 3 Department of Rheumatology, Radboud University Nijmegen Medical Center, Geert-Grooteplein-Zuid 10, 6525 EX, Nijmegen, The Netherlands; 4 Department of Medical Oncology, VU University Medical Center, De Boelelaan 1117, 1081 HV, Amsterdam, The Netherlands; 5 Department of Pathology, VU University Medical Center, De Boelelaan 1117, 1081 HV, Amsterdam, The Netherlands

## Abstract

**Introduction:**

Systemic sclerosis (SSc) complicated by pulmonary arterial hypertension (PAH) carries a poor prognosis, despite pulmonary vascular dilating therapy. Platelet-derived growth factor receptor-β (PDGFR-β) and epidermal growth factor receptor (EGFR) are potential therapeutic targets for PAH because of their proliferative effects on vessel remodelling. To explore their role in SScPAH, we compared PDGFR- and EGFR-mmunoreactivity in lung tissue specimens from SScPAH. We compared staining patterns with idiopathic PAH (IPAH) and pulmonary veno-occlusive disease (PVOD), as SScPAH vasculopathy differs from IPAH and sometimes displays features of PVOD. Immunoreactivity patterns of phosphorylated PDGFR-β (pPDGFR-β) and the ligand PDGF-B were evaluated to provide more insight into the patterns of PDGFR-b activation.

**Methods:**

Lung tissue specimens from five SScPAH, nine IPAH, six PVOD patients and five controls were examined. Immunoreactivity was scored for presence, distribution and intensity.

**Results:**

All SScPAH and three of nine IPAH cases (*P *= 0.03) showed PDGFR-β-immunoreactivity in small vessels (arterioles/venules); of five SScPAH *vs*. two of nine IPAH cases (*P *= 0.02) showed venous immunoreactivity. In small vessels, intensity was stronger in SScPAH *vs*. IPAH. No differences were found between SScPAH and PVOD. One of five normal controls demonstrated focally mild immunoreactivity. There were no differences in PDGF-ligand and pPDGFR-b-immunoreactivity between patient groups; however, pPDGFR-b-immunoreactivity tended to be more prevalent in SScPAH small vasculature compared to IPAH. Vascular EGFR-immunoreactivity was limited to arterial and arteriolar walls, without differences between groups. No immunoreactivity was observed in vasculature of normals.

**Conclusions:**

PDGFR-β-immunoreactivity in SScPAH is more common and intense in small- and post-capillary vessels than in IPAH and does not differ from PVOD, fitting in with histomorphological distribution of vasculopathy. PDGFR-β immunoreactivity pattern is not paralleled by pPDGFR-β or PDGF-B patterns. PDGFR-β- and EGFR-immunoreactivity of pulmonary vessels distinguishes PAH patients from controls.

## Introduction

Systemic sclerosis (SSc) is an autoimmune disease characterized by dysfunction of endothelium, an altered immune tolerance and the deposition of excessive amounts of extra-cellular matrix components in multiple organ systems (reviewed by Gabrielli in [1]). Pulmonary involvement, either lung fibrosis or pulmonary arterial hypertension (PAH), is the leading cause of death in SSc [2]. Patients with SSc are at high risk of developing PAH, with estimated prevalences ranging from 7.9 to 12% [3,4]. SScPAH carries a poor prognosis with three-year patient survival rates of 47 to 56% despite therapy [5-8], although survival has improved when compared with historical series. Still, these survival rates are worse compared to, for example, idiopathic PAH (IPAH). In SScPAH, the clinical benefit from current PAH therapies also compares unfavourably to that of IPAH [9-11], although some have been reported effective [12,13]. SScPAH also differs from IPAH with respect to pulmonary and hemodynamic function [14-17]. Notably, SScPAH typically has lower right ventricular- and pulmonary artery pressures as well as diffusion capacity of the lung for carbon monoxide [6,14,15,17,18]. Pulmonary vasculopathy in SScPAH differs qualitatively from that of IPAH and resembles pulmonary veno-occlusive disease (PVOD), a rare form of PAH, in some instances [19,20]. It seems reasonable to assume that the clinical and histomorphologic differences point to quantitative or even qualitative differences in pathogenetic mechanisms of pulmonary vascular lesions in SScPAH and IPAH.

Growth factor receptors, such as platelet-derived growth factor receptor (PDGFR-β) and epidermal growth factor receptor (EGFR), have been implicated in the pathogenesis of SSc. SSc skin and cultured fibroblasts demonstrate enhanced protein expression of PDGFR-β, and in SSc patients with progressive disease, increased PDGFR-β-plasma levels have been found [21-25]. Imatinib, a dual inhibitor of the tyrosine kinase c-Abl and PDGFR, has been shown to inhibit progression and to induce regression of fibrosis *in vivo *[26]. In addition, increased expression of EGFR in fibroblasts from patients with SSc has been shown [27,28]. Indirect relations with the EGFR signaling system and TGF-b, an important pro-fibrotic mediator in SSc, have been described [27]. In pulmonary hypertension, a role of PDGFR-β and EGFR in the improvement of hemodynamic function has been suggested in animal models [29-31]. It is noteworthy in this context that PDGFR-β plays a role in activation of EGFR [32]. In IPAH patients, increased and activated PDGFR-β has been demonstrated in pulmonary arteries [33]. Moreover, there is anecdotal evidence that inhibition of PDGFR-β is effective in patients with IPAH and in patients with PVOD [34-37]. The role of PDGFR-β and EGFR in SScPAH, however, is as yet unclear.

Here, we examined the presence, localization and intensity of immunostaining for PDGFR-β and EGFR in the pulmonary vasculature of SScPAH, and compared these with IPAH, PVOD, and normal controls. Phosphorylated (p) PDGFR-β and PDGF-B immunoractivity was evaluated to give more insight in activation patterns of PDGFR-β.

## Materials and methods

### Patients

The diagnosis of SScPAH, IPAH and PVOD was verified by reviewing the medical records. Only patients diagnosed with PAH upon right heart catheterization, with a mean resting pulmonary arterial pressure (mPpa) ≥25 mmHg and a pulmonary capillary wedge pressure ≤15 mmHg, were included. The diagnosis of SSc was established by a rheumatologist. SSc patients had to fulfil the preliminary ACR classification criteria for SSc and were classified according to LeRoy *et al. *[38,39]. Patients with restrictive disease as indicated by total lung capacity as a percentage of predicted (TLC%) <70%, vital capacity (VC%) <70% and/or severe fibrosis on HRCT scan were excluded. Lung tissue from five subjects who had died from extra-pulmonary trauma and who had no cardiorespiratory medical history, was used as a control. Histopathological diagnosis of pulmonary vascular disease was confirmed by independent reading by two pathologists (WJM, KG). PVOD was diagnosed based on the presence of a picture of patchy intense capillary congestion in the alveolar parenchyma, and obliterative intimal, loosely textured fibrosis of small veins and venules. PVOD cases did not have arterialised interlobular veins: this is indicative of congestive vasculopathy [40,41].

The cases were collected from the Departments of Pulmonary Diseases and Rheumatology of the VU University Medical Center, Amsterdam and from the Department of Rheumatology of the Radboud University Nijmegen Medical Center, Nijmegen, both in The Netherlands. The study, including the use of archived tissue was approved by the Institutional Review Board on Research Involving Human Subjects of the VU University Medical Center.

### Tissue preparation and immunohistochemistry

Immunohistochemistry was performed on formalin-fixed paraffin-embedded 4 μm sections of lung tissue. All sections were stained in one batch for each marker. Antibodies against PDGFR-β (Cell Signaling Technology, Danvers, MA, USA) and pPDGFR-β (Novus Biologicals, Littleton, CO, USA) were used at dilutions of 1:50 and 1:150, respectively. Active PDGF is built up by polypeptides that form hetero- and homodimers. An antibody specific for the PDGF-B form (Novus Biologicals) was used; it reacts with the PDGF (AB) and PDGF (BB) protein. The dilution used for this antibody was 1:400. For EGFR staining, a monoclonal antibody against EGFR (Novocastra, Newcastle upon Tyne, UK) was used. Immunostaining for the constitutively expressed endothelial marker CD31 (PECAM-1, clone JC70a, Dako, Glostrup, Denmark) served as a reference for the exact localization of PDGFR-b and EGFR staining, as well as for PDGFR-b- and EGFR staining intensity, as staining intensity might be influenced by age of the blocks and duration of fixation. Isotype-matched control-staining was performed with rabbit anti-FITC IgG (Invitrogen, Camarillo, CA, USA). Additional detail on immunostaining is provided in an online data supplement (Additional file 1; figures of isotype-matched control staining: Additional file 2).

### Scoring

Intensity of immunoreactivity was scored semi-quantitatively as absent, mild, moderate and strong on a 0 to 3 point scale. Immunoreactivity was assessed in pulmonary arteries, arterioles, capillaries, venules and veins, and, where applicable, in intima, media and adventitia. Arteries were identified by their accompanying bronchiole and the presence of a lamina elastica interna and externa. Vessels were identified as arteriole when their parent artery could be identified. In case arterioles or venules could not be distinguished by their anatomical localisation, they were collectively designated as "small vessels". Veins were identified in case they were located in interlobular septa, and venules in case they could be anatomically deduced from a draining vein. Intimal fibrosis was recognizable by Elastica von Gieson-stained slides.

The overall distribution of immunoreactivity in vessels was scored as focal, multifocal or widespread, with reference to the type of vessel and micro-anatomical localization. In case of pPDGF-β and PDGF-B, positively stained cells were assessed as 0 to 25%, 25 to 50%, 50 to 75% and >75%. Staining was designated as focal if 25%, multifocal if 25 to 75% and widespread if more than 75% of the cells were positively stained. Scoring took place by two independent readers (KG, MJO) blinded to the clinical diagnoses. Discrepant scores were reviewed to reach consensus. In none of the cases was there disagreement.

### Statistics

SPSS 12.0 software package (Chicago, IL, USA) was used for statistical analyses. The Kruskal-Wallis test was used for comparison of means concerning demographic-, pulmonary function- and hemodynamic parameters. For the comparison of the presence and of the intensity of immunoreactivity, Fisher's Exact test was used to compare non-parametric data between groups. A *P*-value < 0.05 was considered statistically significant. Other parameters were analysed descriptively due to lack of statistical power.

## Results

Lung tissue samples from five SScPAH, nine IPAH, six PVOD patients and five controls were collected. Samples had been obtained at autopsy (*n *= 17), open lung biopsy (*n *= 5; one SScPAH patient, four PVOD patients) or at lung explantation (*n *= 3; one SScPAH, one IPAH and one PVOD patient). Patient characteristics are shown in Table 1. The SSc patients were classified as having the limited cutaneous form of the disease [40]. The groups did not differ significantly with respect to mean age. None of the patients outside the SSc group had been diagnosed with systemic sclerosis. The hemodynamic parameters, listed in Table 2, were not significantly different between the SScPAH, IPAH and PVOD groups. CD31 staining intensity varied only marginally among cases.

**Table 1:** General patient characteristics

	SScPAH *N *= 5	IPAH *N *= 9	PVOD *N *= 6	Control *N *= 5
Age, yrs	51 (32 to 60)	53 (23 to 59)	33 (23 to 59)	33 (24 to 76)
Male/Female (n)	1/4	2/7	3/3	5/0
Antibody (Ab) profile	anti-centromere: 5 (100%)	0	0	
Disease duration of PAH at time of death/biopsy (yrs)	1 (0.1 to 4.0)	2.6 (0.8 to 9.0)	1.9 (0.08 to 5.0)	
Therapy at time of death/biopsy				
Monotherapy:				
prostacycline	3	6	4	
PDE-5 inhibitor	0	1	0	
ERA	0	1	2	
Combination therapy:				
Prostacycline+ERA+		0	0	
PDE-5 inibitor (n)	1			
ERA+PDE-5 inhibitor (n)	1	0	0	
ERA+prostacycline (n)	0	1	0	
Limited cutaneous SSc, n (%)	5 (100%)			
SSc disease duration, yrs *	2 (1 to 34)			
Raynaud phenomenon duration, yrs	13 (1 to 40)			

Values expressed as median (range) or otherwise as stated. * Since first non-Raynaud symptom, at time of diagnosis of pulmonary arterial hypertension.

ABS, atrial balloon septostomia; ERA, endothelin receptor antagonist; IPAH, idiopathic PAH; PDE-5, phosphodiesterase 5; PVOD, pulmonary veno-occlusive disease; SScPAH, systemic sclerosis-associated pulmonary arterial hypertension (PAH).

**Table 2:** Haemodynamic parameters

	SScPAH *N *= 5	IPAH *N *= 9	PVOD *N *= 6
mRpa, mmHg	10 (6 to 18) (*n *= 4)	11(7 to 23) (*n *= 5)	10 (5 to 15) (*n *= 5)
sPpa, mmHg	78 (70 to 101) (*n *= 5)	100 (62 to 109) (*n *= 9)	76 (58 to 100) (*n *= 6)
mPpa, mmHg	55 (43 to 71) (*n *= 5)	55 (43 to 76) (*n *= 7)	49 (45 to 70) (*n *= 6)
PCWP,mmHg	9 (5 to 12) (*n *= 5)	7 (1 to 12) (*n *= 7)	5 (5 to 11) (*n *= 6)
PVR, dynes·s · cm^-5^	1054 (627 to 3278) (*n *= 4)	957 (916 to 1587) (*n *= 6)	750 (479 to 1483) (*n *= 7)
CI, l/min·m^2^	2.2 (1.1 to 2.9) (*n *= 7)	2.4 (1.3 to 3.2) (*n *= 8)	2.6 (1.9 to 3.1) (*n *= 6)
FEV1, %	92 (77 to 97) (*n *= 4)	80 (56 to 111) (*n *= 8)	82 (74 to 95) (*n *= 6)
TLC, %	87 (81 to 115) (*n *= 3)	94 (78 to 113) (*n *= 9)	90 (81 to 105) (*n *= 6)
VC, %	99 (88 to 120) (*n *= 4)	87 (58 to 119) (*n *= 8)	*
TLCO, %	35 (26 to 57) (*n *= 4)	60 (52 to 90) (*n *= 8)	49 (20 to 69) (*n *= 6)

Values expressed as median (range) or otherwise as stated. * not available

CI, cardiac index; FEV1 %, percentage predicted of forced expiratory volume; IPAH, idiopathic PAH; mRpa, mean right atrial pressure; PCWP, pulmonary capillary wedge pressure; PVOD, pulmonary veno-occlusive disease; PVR, pulmonary vascular resistance; sPpa, mPpa, systolic, mean pulmonary artery pressures; SScPAH, systemic sclerosis-associated pulmonary arterial hypertension (PAH); TLC %, percentage of predicted total long capacity; TLCO %, percentage predicted of transfer factor of the lung for carbon monoxide; VC, percentage predicted of vital capacity.

### PDGFR-β immunoreactivity

**Figure 1:**
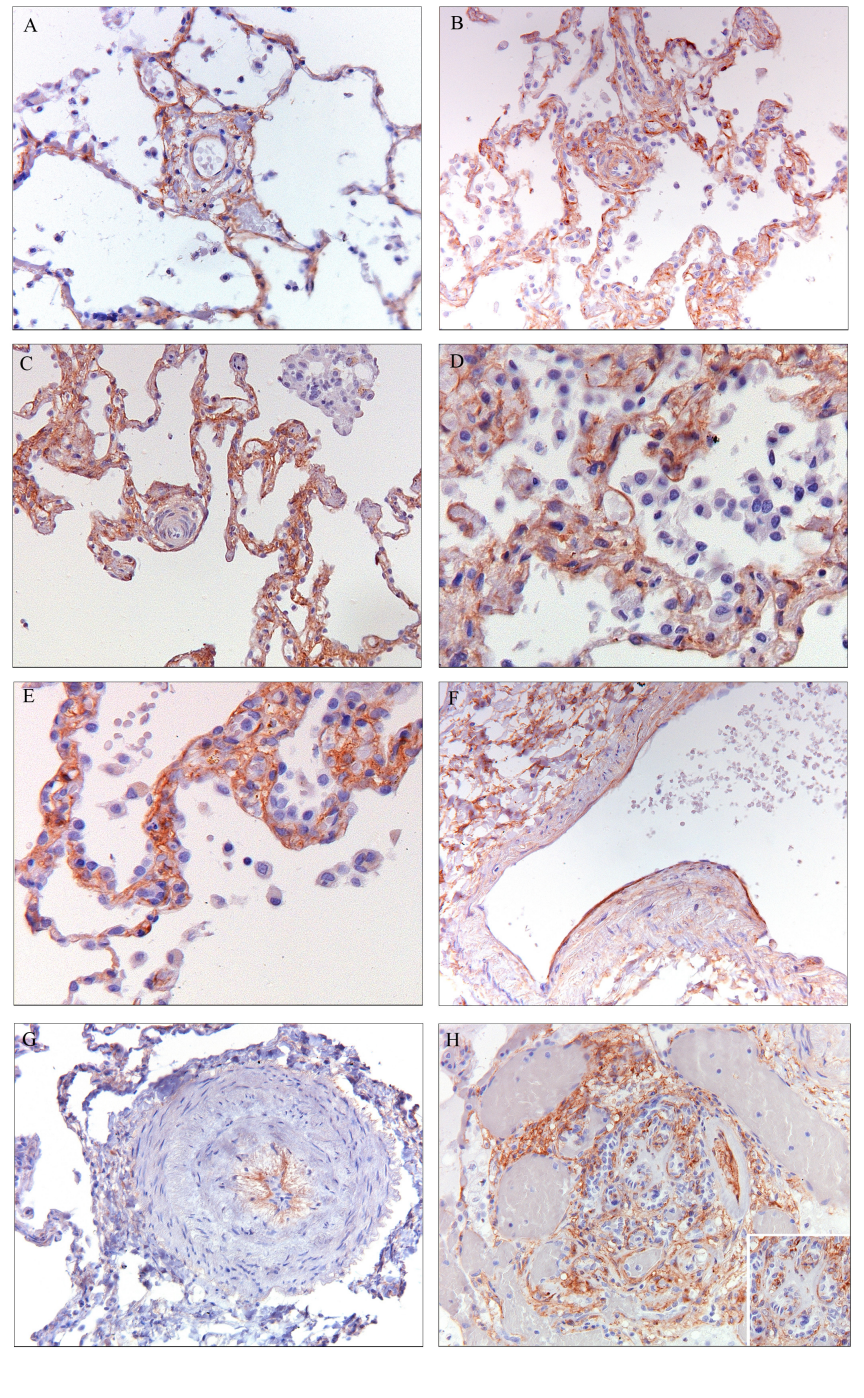
**Plateled-derived growth factor receptor (PDGFR)-β immunoreactivity in pulmonary vessels in SScPAH, IPAH and PVOD**. (Original magnification 200×). **A**) PDGFR-β immunoreactivity in the endothelial layer of a small corner vessel without fibrosis in SScPAH. Note the epithelial immunoreactivity at the alveolar attachments of the corner vessel, serving as an internal positive control. **B**) PDGFR-β immunoreactivity in the endothelium of a small corner vessel with fibrosis in SScPAH, and in the (congested) capillary endothelium. **C**) Absence of PDGFR-β immunoreactivity in the endothelial layer of a small corner vessel with intimal fibrosis, in an area of capillary congestion, in PVOD. There is epithelial immunoreactivity at the alveolar attachments of the corner vessel. **D**) PDGFR-β immunoreactivity in endothelial cells of capillaries in a SScPAH patient. **E**) Intense PDGFR-β endothelial immunoreactivity in an area of congestion in a PVOD patient. **F**) PDGFR-β endothelial immunoreactivity in a vein in a SScPAH patient. **G**) PDGFR-β immunoreactivity in the basal side of the endothelium of an arteriole with intimal fibrosis and media hyperplasia in an IPAH patient. **H**) PDGFR-β immunoreactivity in a plexiform lesion in an IPAH patient; immunoreactivity of basal side of the endothelial cells (insert) in the glomeruloid lesion, surrounded by vein-like branches (dilatation lesions).

In SScPAH, PDGFR-β immunoreactivity was present in the complete spectrum of the pulmonary vasculature, in vessels both with and without intimal fibrosis. PDGFR-β was expressed focally in the adventitia and media of axial arteries and arterioles. In the intimal layer of the small vessels, all SScPAH patients demonstrated, albeit focally, immunoreactivity (Figure 1A, B). In the capillaries, PDGFR-β immunoreactivity was widespread in each of the five SScPAH patients (Figure 1A, B, D). This immunoreactivity was present in areas with and without congestion. At venular-venous level, in four out of five SScPAH patients a mild, focal PDGFR-β immunoreactivity was observed in the intima (Figure 1F).

**Figure 2:**
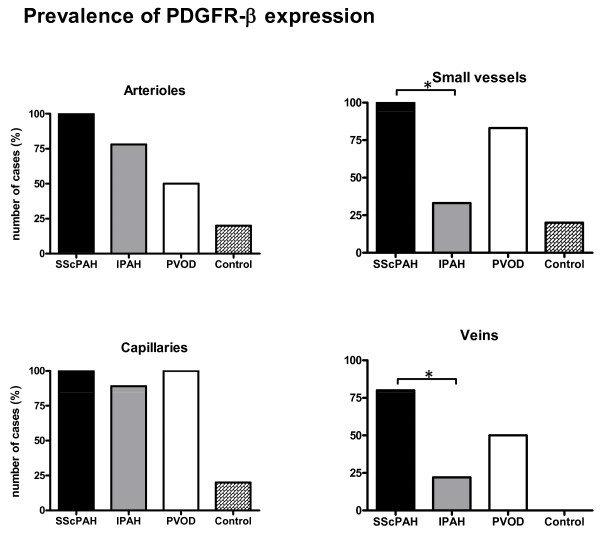
**Number of cases with plateled-derived growth factor receptor (PDGFR)-β-immunoreactivity in the intima of pulmonary vessels in SScPAH, IPAH, PVOD, controls**. Small vessels: those arterioles and/or venules that cannot be distinguished based on their anatomical localisation. * *P *< 0.05

**Figure 3:**
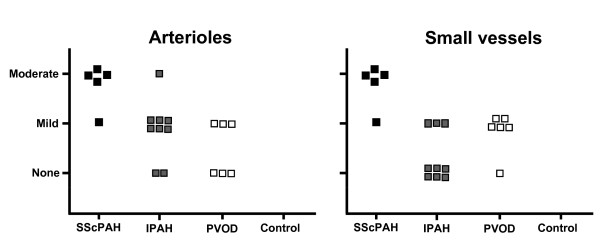
**Intensity of plateled-derived growth factor-receptor (PDGFR)-β-immunostaining in the intima of pulmonary vessels in SScPAH, IPAH, PVOD and controls**. A: arterioles. B: small vessels.For the pooled arterioles and small vessels, intensity in SScPAH was stronger than in IPAH (*P *= 0.02). Each image represents one case. Small vessels: those arterioles and/or venules that cannot be distinguished as such based on their anatomical localisation.

In IPAH, PDGFR-β immunoreactivity of the intimal and adventitial layers of the arteries and the arterioles was focally observed (Figure 1G). Only three out of nine IPAH patients revealed a focal immunoreactivity of the intima in small vessels. The prevalence was significantly lower as compared with SScPAH (*P *= 0.03) (Figure 2). Moreover, intensity of immunoreactivity in the pooled arterioles and small vessels was weaker in IPAH than in SScPAH (*P *= 0.02) (Figure 3). The interlobular veins and venules were focally, mildly stained, but, again, in lower frequency in IPAH than in SScPAH (*P *= 0.02). Capillaries were PDGFR-β positive in eight out of nine IPAH cases. Plexiform lesions, observed in eight out of nine IPAH cases, showed mild PDGFR-β positivity: in some cases there was only immunoreactivity of endothelium while in other lesions there was immunoreactivity of endothelial and subendothelial stromal cells, with thin lines of positive immunoreactivity demarcating the basal side of endothelial cells (Figure 1H).

Two out of six PVOD cases demonstrated intimal immunoreactivity in the entire spectrum of the pulmonary vasculature. Pre-capillary intimal and adventitial immunoreactivity with a mild intensity was observed focally in three PVOD patients. In five out of six patients, a focal immunoreactivity of small vasculature intima was observed (Figure 2). Capillary immunoreactivity, present in areas with and without congestion, was widespread (Figure 1E), with an intensity from mild to strong. No differences were found in prevalence, localisation or intensity of PDGFR-β in the PVOD group when compared to the SScPAH or the IPAH group.

In the control group, only one subject demonstrated, focally, a mild PDGFR-β immunoreactivity in pre-capillary vessels and capillaries, but not in post-capillary vessels. Figures of control slides are added in an online data supplement (Additional file 3).

### pPDGFR-β immunoreactivity

**Figure 4:**
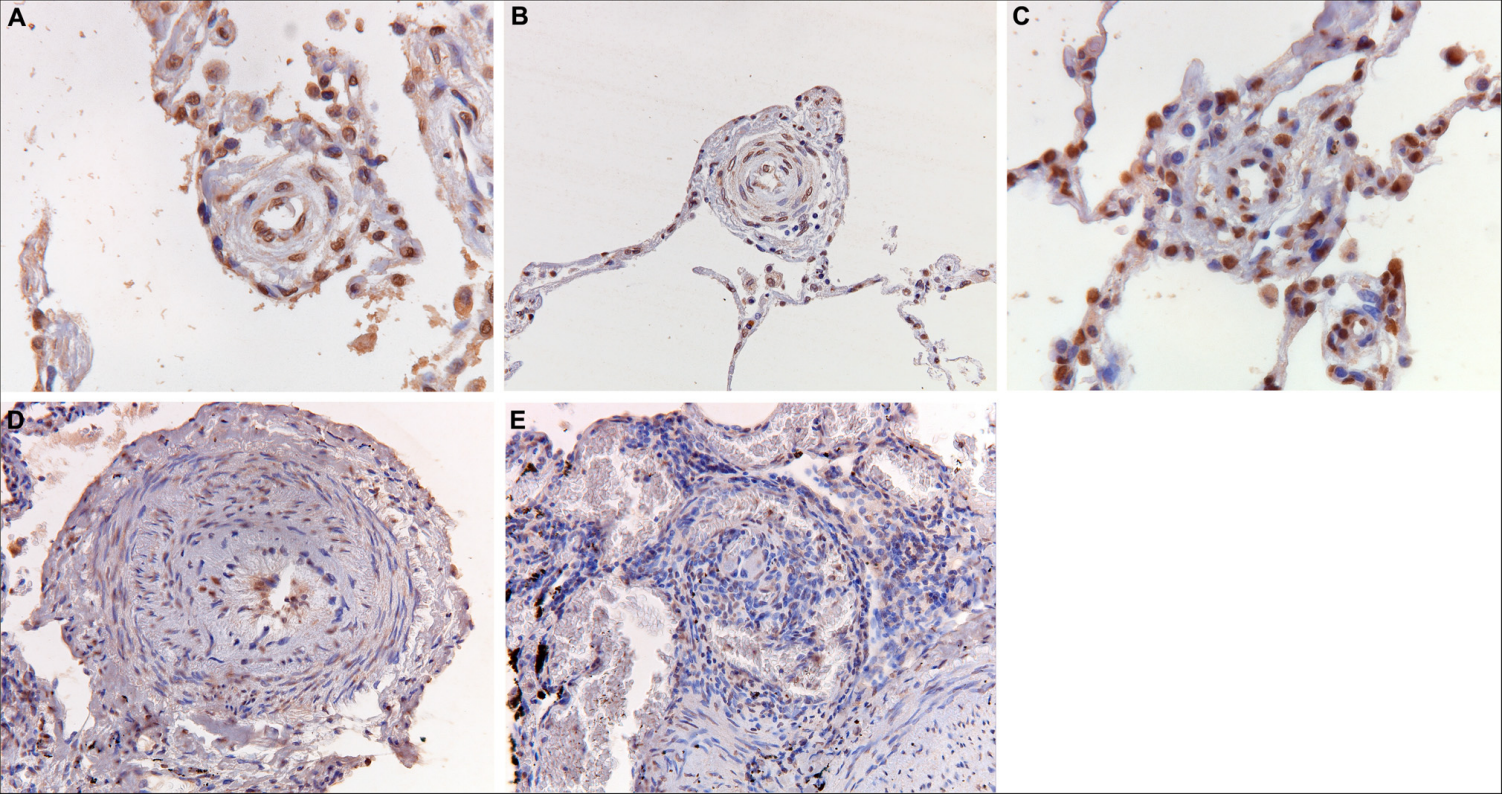
**Phosphorylated plateled-derived growth factor receptor (pPDGFR)-β immunoreactivity in pulmonary vessels in SScPAH, IPAH and PVOD**. (Original magnification 200×) **A**) pPDGFR-β immunoreactivity in endothelial cell nuclei in a small vessel with intimal fibrosis in a patient with SScPAH. **B**) pPDGFR-β immunoreactivity in cell nuclei in the intima of an arteriole with concentric laminar intimal fibrosis in a patient with SScPAH (see also 6B). **C**) pPDGFR-β immunoreactivity in cell nuclei of a small vessel and capillaries in a PVOD patient. **D**) pPDGFR-β immunoreactivity in the basal side of the endothelium, the thickened intima and in smooth muscle cells of the hyperplastic media of an arteriole of an IPAH patient (see also Figures 1G and 6D). **E**) pPDGFR-β immunoreactivity in stroma and endothelium of a plexiform lesion in an IPAH patient. Lower right quadrant parent artery. In the center a glomeruloid lesion surrounded by vein-like branches (dilatation lesions).

**Figure 5:**
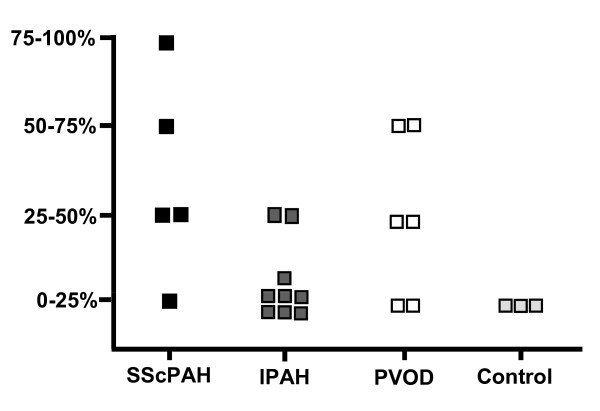
**Amount of phosphorylated plateled-derived growth factor receptor (pPDGFR)-β-positively immunostained cells in intima of the small vessels in SScPAH, IPAH, PVOD and controls**. With a cut off of 25% cell staining, a trend was shown (*P *= 0.09) in favor of more positive cell immunoreactivity in small vasculature in SScPAH patients *vs*. IPAH patients. Each image represents one case.

pPDGFR-β was present in the pre-, post- and capillary pulmonary vasculature in all patient groups. In Figure 4, representative pictures of pPDGFR-β immunoreactivity are displayed. Staining was predominantly present in the nuclei of the cells. In the pre-capillary vessels, immunoreactivity was observed in the smooth muscle cells of the media in all patient groups. Intimal cells were also positively stained in the diseased groups. This was seen in vessels with and without intimal fibrosis. With a cut off of 25% cell staining, a trend was shown (*P *= 0.09) in favor of more positive cell immunoreactivity in small vasculature in SScPAH patients *vs*. IPAH patients (Figure 5). The capillaries demonstrated immunoreactivity in all patients with no difference between the groups. Post-capillary staining was found in the intimal layers of all SScPAH and PVOD patients and in six out of nine IPAH patients, without quantitative differences. Bronchioles in all patients and controls uniformly demonstrated pPGFR-β immunoreactivity in the nuclei of the basal layers of the epithelium and as such served as a positive internal control (Figure 4F). Controls showed staining in the whole pulmonary vascular tree; however, this was a focal staining, with cell counts not exceeding 25%.

### PDGF-B immunoreactivity

**Figure 6:**
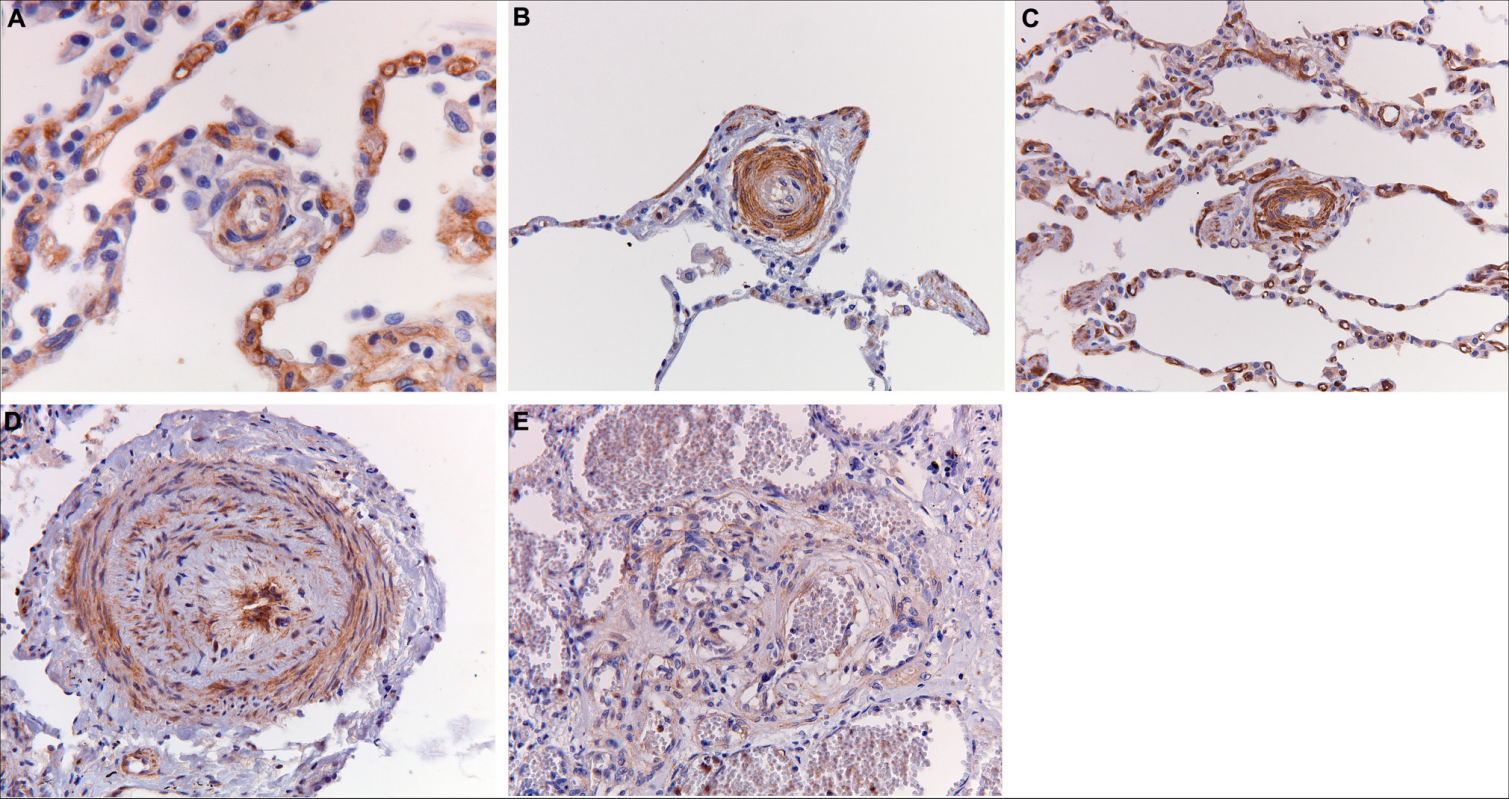
**Plateled-derived growth factor (PDGF) B immunoreactivity in pulmonary vessels in SScPAH, IPAH and PVOD**. (Original magnification 200×). **A**) PDGF-B immunoreactivity in small vessel and capillaries in a patient with SScPAH. **B**) PDGF-B immunoreactivity in the intima layers of an arteriole with concentric laminar intimal fibrosis in a patient with SScPAH (see also 4B). **C**) PDGF-B immunoreactivity in a small vessel and (congested) capillaries in a PVOD patient. **D**) PDGF-B immunoreactivity in the basal side of the endothelium, the thickened intima and in smooth muscle cells in the hyperplastic media of an arteriole in a IPAH patient. **E**) PDGF-B immunoreactivity in stroma and endothelium of a plexiform lesion in an IPAH patient. In the center a glomeroid lesiom surrounded by vein-like branches (dilatation lesions).

PDGF-B demonstrated immunoreactivity in the complete spectrum of the pulmonary vascular tree in all patient groups. Representative pictures of PDFG-B are displayed in Figure 6. One IPAH patient failed to demonstrate immunoreactivity in the capillaries and one PVOD patient did not show PDGF-B staining in the post-capillary vessels. PDGF-B staining was remarkably widespread in the axial arteries and arterioles, both in media and intima. The small vessels demonstrated a widely spread distribution of immunoreactivity. The capillaries were mostly stained in a multifocal to widespread fashion, as were the venules and veins. Staining was more widespread as compared with PDGFR-β and pPDGFR-β, in all patient groups. All the plexiform lesions in the IPAH patients demonstrated immunoreactivity of pPDGFR-β and PDGF-B in both the endothelial and stromal cells. As in pPDGFR-β, PDGF-B was also uniformly positively stained in the observed bronchioles in all subjects, and this yielded a positive internal control.

Controls showed pPDGFR-β- and PDGF immunoreactivity in the pulmonary vessels, however, this was a focal, nonuniform staining.

### EGFR immunoreactivity

**Figure 7:**
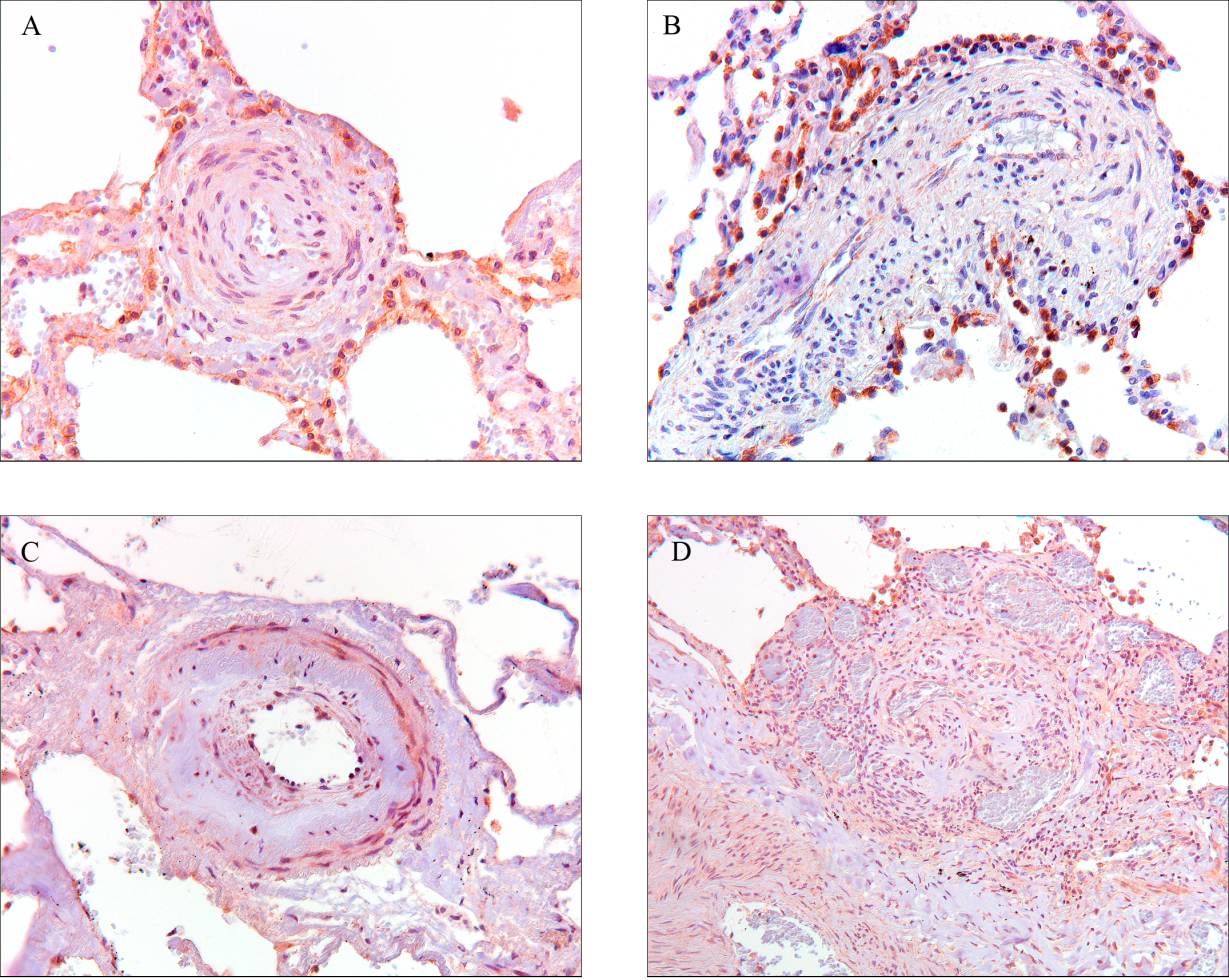
**Epidermal growth factor receptor (EGFR) immunoreactivity in pulmonary vessels in SScPAH, IPAH and PVOD**. (Original magnification 200×). **A**) EGFR expression of alveolar epithelium surrounding a parenchymal arteriole (corner vessel) in a SScPAH patient, showing weak immunoreactivity of the media and endothelium. **B**) Weak EGFR immunoreactivity of endothelial cells in a SScPAH patient. **C**) EGFR expression in media and fibrotic intimal layer of an axial artery of a SScPAH patient. Left lower quadrant: parent artery. Center: glomeruloid lesion, surrounded by dilatation lesions (original magnification 100×). **D**) Plexiform lesion in an IPAH patient showing weak stromal EFGR expression.

EGFR was positive in the basal cell layers of the bronchial epithelium, alveolar epithelial cells and type II pneumocytes in all patient and control cases, serving as an internal control. Interestingly, focal areas of positively immunoreactivity type II pneumocytes were found to surround the pre-capillary vessels (Figure 7A, B) in the patient cases and in one control case. No differences in the prevalence of this phenomenon between the patient groups were observed. Capillaries surrounded by EGFR-expressing pneumocytes were observed in all SScPAH patients, in five out of nine IPAH patients and in two out of six PVOD patients.

**Figure 8:**
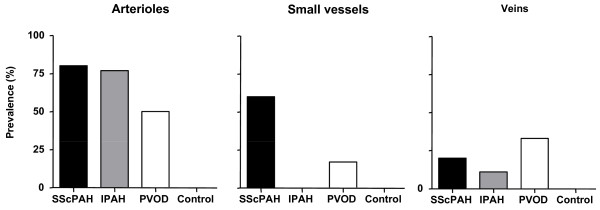
**Number of cases with positive immunostaining for epidermal growth factor receptor (EGFR) in the intima of pulmonary vessels**. **A**: arterioles, **B**: Small vessels, **C**: veins) in SScPAH, IPAH, PVOD and normal controls. No staining was observed in the capillaries. Small vessels: those arterioles and/or venules that cannot be distinguished as such by their anatomical localisation

EGFR immunoreactivity was focal and weak in the pulmonary hypertension groups (Figure 7A-C) and was observed mostly in media and intima of the pulmonary vessels. No differences in immunoreactivity prevalence (Figure 8), intensity or distribution between the pulmonary hypertension groups were observed. Most plexiform lesions demonstrated a weak immunoreactivity of EGFR, which appeared to be located in subendothelial stromal cells (Figure 7D). No immunoreactivity of pulmonary vessels was observed in the control cases.

## Discussion

This study demonstrates the presence of PDGFR-b immunoreactivity in the entire pulmonary vascular bed of SScPAH patients, with a different staining pattern as compared to IPAH. There were no differences in PDGFR-β immunoreactivity between SScPAH and PVOD. PDGFR-β immunoreactivity was more prevalent and intense in the PAH groups than in controls. There was a trend towards more pPDGFR-β-positively stained cells in SScPAH small vasculature as compared with IPAH. EGFR was minimally present in the pulmonary vasculature of SScPAH, IPAH and PVOD, without differences between the groups. No EGFR immunoreactivity was observed in the pulmonary vasculature of controls.

This is the first study to explore PDGFR-β- and EGFR immunoreactivity in lung vasculature in SScPAH. PDGFR-β is implicated in SSc disease [21-26]. In IPAH, Perros *et al. *demonstrated PDGFR-b, pPDGFR-b and PDGF-A and -B expression and activity in remodelled small pulmonary arteries and plexiform lesions [33]. In pulmonary capillary haemangiomatosis, an entity that shows overlap with both PVOD [42] and SScPAH [19], up-regulation of PDGF-B and PDGFR-ß genes has been shown in distended capillaries [43]. The present study supports these findings and extends them by showing the presence of PDGFR-ß immunoreactivity in SScPAH. The different immunoreactivity pattern in the pulmonary vasculature compared to IPAH fits in with the distinctive distribution of vascular lesions in SScPAH. This might implicate a role for PDGFR-β in small vessel intimal remodeling in SScPAH.

EGFR expression in human pulmonary vasculature affected by SSc or SScPAH has not been previously reported. We demonstrate EGFR expression, albeit mild and focal, in human pulmonary vasculature of SScPAH, IPAH and PVOD. Dahal *et al. *[31] failed to show a difference in EGFR expression in lungs of patients with end-stage IPAH and normal controls. This apparent discrepancy compared to the present study may be explained by patient selection, by the use of tissue obtained at lung transplantation and by the evaluation of whole lung tissue by Dahal *et al.*

The inherent drawback of using archival tissue from different laboratories is shared by other studies. Differences in preparation and in storage time may have an unknown influence on the quantity or quality of immunoreactivity. However, care was taken to limit the influence of age of paraffin blocks, and preparation procedures such as fixation time on epitope availability, by using the constitutive expression of CD31 as a positive control within each case. Moreover, the uniform positive immunoreactivity of bronchiolar epithelium in pPDGFR-β, PDGF-B and EGFR samples served as an internal positive control.

Antibodies directed at different epitopes than the ones we used for our experiments, might generate different results. This, in combination with differences in antigen blocking steps, might explain why we did not detect PDGFR-b immunoreactivity in the media of pulmonary arteries in the IPAH group, in contrast to Perros *et al. *[33] However, we did demonstrate PDGFR-β, pPDGFR-β and PDGF-B immunoreactivity in smooth muscle cells and endothelial cells of constrictive pulmonary arteries and plexiform lesions, which is in concordance with Perros *et al. *[33] As immunohistochemical immunoreactivity demonstrates the presence but not the activity of (p)PDGFR-b, PDGF-B and EGFR, further studies are needed to further support the rationale for the use of receptor antagonists in SScPAH. The small sample size limits the interpretation of the results. However, only thoroughly characterized unequivocal cases of SScPAH, IPAH and PVOD were included, so as to reduce overlap. As histopathological information on well-characterized SScPAH patients is scarce, the results obtained here provide valuable exploratory information. However, they underscore the need for sampling of suitable tissue specimens in these patient groups for future research, also into receptor functionality studies. The majority of the PVOD samples were biopsies, while the samples from the SScPAH and IPAH group were derived from autopsy material. We cannot exclude some influence on results, as there is no knowledge on post-mortem behaviour of the (p)PDGFR-β and PDFG-B. Another influencing factor might be the fact that the biopsy group does not necessarily represent end-stage disease, in contrast to the explanation- and autopsy samples.

How do we interpret these results? The pattern of immunoreactivity of PDGFR-β and probably pPDGFR-β in SScPAH, IPAH and PVOD follows the distinct patterns of histomorphologic vasculopathy between these disease groups [20]. The specific role of PDGFR in SScPAH vascular remodeling is further supported by either PDGF or PDGFR autoantibodies [44]. Such antibodies may induce signaling pathways, which eventually may lead to local intimal fibrosis. No differences in the small vessel- and post-capillary vasculature were seen between SScPAH and PVOD. As PVOD-like changes may be seen in SScPAH pulmonary vasculature [19,20] it can be speculated that SScPAH and PVOD share activation of PDGFR-b as a pathophysiologic determinant.

The observation of PDGFR-β immunoreactivity, in both affected and non-affected vessels, might be interpreted as pointing towards longstanding pathogenetic involvement. pPDGFR-β and PDGF-B showed immunoreactivity in the pulmonary vasculature of the diseased patient group, with an increased prevalence as compared to controls. This supports the pathogenetic role of the PDGFR-β pathway in PAH. However, this study neither demonstrated clear parallels in staining patterns between PDGFR-β and pPDGFR-β nor PDGF-B in the SScPAH group. This might be explained by transactivation of PDGFR-β, resulting in phosphorylation of the PDGFR-β [45]. The extent of involvement of the PDGFR-β- pPDGFR-β-signalling pathway in PAH pathogenesis and whether the role of this pathway is different in SScPAH as in IPAH, will need to be investigated in functional studies.

PDGFR-β can be inhibited by imatinib, a TKR inhibitor that also has specificity for the Abl-related gene protein in the tyrosine fusion protein Bcr-Abl and c-kit. The effect of imatinib in SSc pathogenesis might be enhanced by its inhibitory effect on c-Abl, which is important for the induction of extracellular matrix components via TGF-β signaling [46,47]. TGF-β is among the most important pro-fibrotic SSc-mediators [67]. This, together with the findings in the present study support the rationale for PDGFR-b targeted therapy in SScPAH. The effects of such therapy might extend to EGFR via transactivation by PDGFR-b, leading to altered signalling of the EGFR [48].

PDGFR-β, its ligand and its phosphorylated state and EGFR were observed in plexiform lesions of IPAH patients. Their active participation in plexiform lesion formation remains speculative, but Perros *et al. *[33] demonstrated immunoreactivity of PDGFR-β, PDGF-BB and phosphorylated PDGFR-β in endothelium-lined channels, fitting in with the findings in the present study. This is the first report of EGFR expression in plexiform lesions. It can be speculated that EGFR features in their formation: Tuder *et al. *demonstrated that endothelial cells in plexiform lesions expressed the transcription factor units HIF-1a and HIF-1b [49]. In cancers, HIF-1 participates in the activation of autocrine signaling pathways involving TGF-a/EGFR and EGF-2/IGF-1R, which promote cell survival and proliferation [50,51]. As the role of plexiform lesions in haemodynamic alterations occurring in PH is unknown, it is uncertain as to whether treatment aimed at their growth factor receptors will be effective in IPAH.

## Conclusions

We demonstrated that the PDGFR-b immunoreactivity pattern in SScPAH differs from that in IPAH, whereas no differences were observed between SScPAH and PVOD. This is in line with differences in distribution and morphologic characteristics of vasculopathy between the disease groups. This might implicate that PDGFR-β activation plays a role in pulmonary hypertension, which is supported by the presence of its phosphorylated state and the PDGFR-B ligand. The mild immunoreactivity of EGFR in PAH vasculature as compared to its total absence in controls might be an indication of its pathogeneity in PAH, too. This study supports the notion that PDGFR-inhibiting therapy may be effective in the treatment of PAH and of SScPAH in particular, and that multikinase inhibitors deserve consideration as an option in future treatment strategies in pulmonary arterial hypertension.

## Abbreviations

EGFR: epidermal growth factor receptor; IPAH: idiopathic pulmonary arterial hypertension; PAH: pulmonary arterial hypertension; PDGFR-β: plateled-derived growth factor receptor-β; p PDGFR-β: phosphorylated plateled-derived growth factor receptor-β; PVOD: pulmonary veno-occlusive disease; SScPAH: systemic sclerosis-associated pulmonary arterial hypertension.

## Competing interests

The authors declare that they have no competing interests.

## Authors' contributions

MJO designed the manuscript, collected the patient data and material, performed the immunohistochemistry experiments, scored the immunoreactivity, performed the statistical analysis, analyzed the data and drafted the manuscript. AB and AEV designed the manuscript, analyzed the data and drafted the manuscript. LSH performed immunohistochemistry experiments. MCV collected the patient data and material and analyzed the data. AVN designed the manuscript and drafted the manuscript. WJM analyzed the data and drafted the manuscript. BACD and EFS designed the manuscript and drafted the manuscript. KG designed the manuscript, scored the immunoreactivity, analyzed the data and drafted the manuscript.

## Supplementary Material

Additional file 1 **Tissue preparation and immunohistochemistry**. Additional file concerning detailed description of tissue preparation and immunohistochemistry Click here for file

Additional file 2 **Immunohistochemistry in normal controls**. Additional file with representative figures of immunoreactivity of PDGFR-β, p PDGFR-β, PDGF-B and EGFR in normal control subjects. **A**. PDGFR-b immunoreactivity in a small vessel of a healthy control. **B**. pPDGFR-b immunoreactivity in a small vessel of a healthy control. **C**. PDGF AB/BB immunoreactivity in an axial artery and bronchiole of a healthy control. **D**. EGFR in a small vessel of a healthy control. Click here for file

Additional file 3 **Isoptype-matched control staining**. additional file with representative figures of isoptype-matched control stainings of SScPAH-, IPAH- and PVOD-staining Click here for file
