# Supplementary material for: Platelet-derived growth factor receptor-β and epidermal growth factor receptor in pulmonary vasculature of systemic sclerosis-associated pulmonary arterial hypertension versus idiopathic pulmonary arterial hypertension and pulmonary veno-occlusive disease: a case-control study
Source: Arthritis Res Ther. 2011 Apr 14;13(2):R61. doi: 10.1186/ar3315 (PMC3132056; doi:10.1186/ar3315)
Supplement: Additional file 1 — Tissue preparation and immunohistochemistry. Additional file concerning detailed description of tissue preparation and immunohistochemistry [file ar3315-S1.DOC]

# Tissue preparation and immunohistochemistry

Immunohistochemistry was performed on formalin-fixed paraffin-embedded 4μm sections of lung tissue. All sections were stained in one batch for each marker. After deparaffination in xylene and rehydration through graded alcohols, slides were rinsed with phosphate buffered saline (PBS). Endogenous peroxidase was blocked with 0.3% hydrogen peroxidase. For PDGFR-β, pPDGFR-β and PDGF-B, antigen retrieval was done by boiling in citrate buffer (10 mM at pH 6.0); for EGFR staining, antigen retrieval took place in Tris/EDTA buffer (10 mM, pH 9.0) by treatment in respectively autoclave or microwave. Rabbit monoclonal antibody against PDGFR-β (clone 28E1; Cell Signaling Technology, Danvers, MA, USA) was used at a dilution of 1:50, followed by biotinylated swine anti-rabbit antibody (Dako, Glostrup, Denmark), and avidin-biotin-peroxidase complex (Vectastain ABC Kit; Vector). Horseradish peroxidase (HRP) activity was detected using H_2_O_2_ as substrate and 3,3
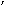
-diaminobenzidine tetrahydrochloride as dye. For pPDGFR-β staining, rabbit polyclonal antibody against pPDGFR-β (phosphor Tyr 751) (Novus Biologicals, Littleton, CO, USA) was used at a dilution of 1:150. Immunoreactivity of PDGF-B was evaluated by the use of rabbit polyclonal antibody against the 101-116aa human epitope of human PDGF-B (Novus Biologicals, Littleton, CO, USA), at a dilution of 1:400. pPDGFR-β and PDGF-B antibody staining was followed by incubation with Envision HRP rabbit/mouse polymer (Dako, Glostrup, Denmark). For EGFR staining, monoclonal antibody against EGFR (Novocastra, Newcastle upon Tyne, UK) was used at a dilution of 1:25, followed by incubation with **Powervision** post-antibody blocking solution (Immunovision Technologies, Daly City, CA, USA). The slides were then incubated with **Powervision** polymerised HRP conjugates (Immunovision Technologies, Daly City, CA, USA) and the sections were stained with 3,3'-diaminobenzidine tetrahydrochloride as chromogen. Isotype-matched control staining was performed with rabbit polyconal anti-FITC antibody (ZF24, Invitrogen, Camarillo, CA,USA) at a dilution of 1:37.5 and 1:375 (figure 2S). Immunostaining for the constitutively expressed endothelial marker CD31 (PECAM-1, clone JC70a, Dako, Glostrup Denmark) served as a reference for the exact localization of PDGFR-β and EGFR staining, as well as for PDGFR-β- and EGFR staining intensity, as staining intensity might be influenced by age of the blocks and duration of fixation. All sections were counterstained with Mayer’s haematoxylin.

**Figure 2S.** Isoptype-matched control staining (original magnification x200)

A. Arteriole with intimal fibrosis in a patient with SScPAH

B. Arteriole with intimal fibrosis and media hyperplasia in an IPAH patient

C. Plexiform lesion in an IPAH patient (original magnification x100)

D. Small vessel in an area of congestion in a PVOD patient.
