# Supplementary figures and images for: Platelet-derived growth factor receptor-β and epidermal growth factor receptor in pulmonary vasculature of systemic sclerosis-associated pulmonary arterial hypertension versus idiopathic pulmonary arterial hypertension and pulmonary veno-occlusive disease: a case-control study
Source: Arthritis Res Ther. 2011 Apr 14;13(2):R61. doi: 10.1186/ar3315 (PMC3132056; doi:10.1186/ar3315)

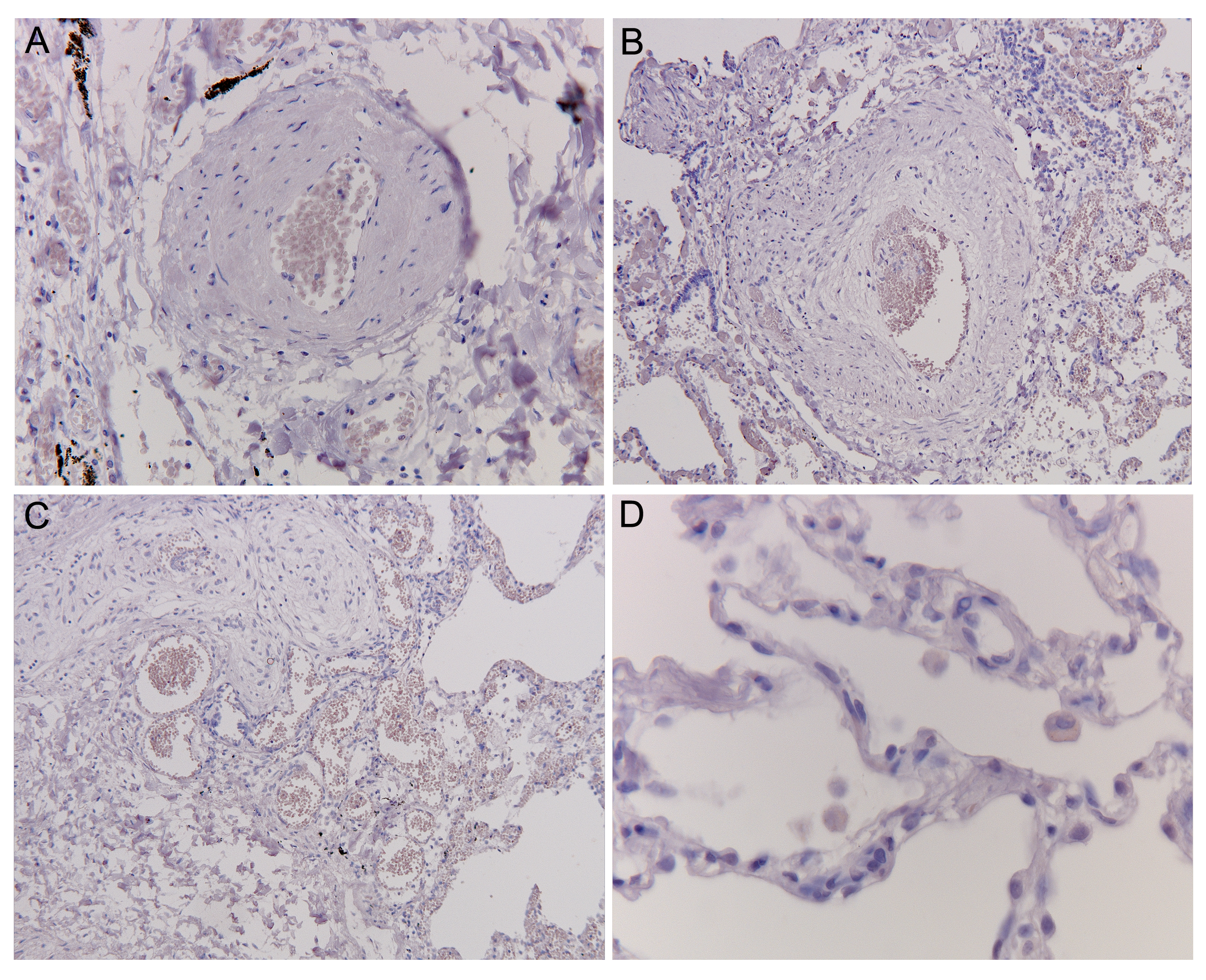

Supplement: Additional file 2 — Immunohistochemistry in normal controls. Additional file with representative figures of immunoreactivity of PDGFR-β, p PDGFR-β, PDGF-B and EGFR in normal control subjects. A. PDGFR-b immunoreactivity in a small vessel of a healthy control. B. pPDGFR-b immunoreactivity in a small vessel of a healthy control. C. PDGF AB/BB immunoreactivity in an axial artery and bronchiole of a healthy control. D. EGFR in a small vessel of a healthy control. [file ar3315-S2.TIFF]

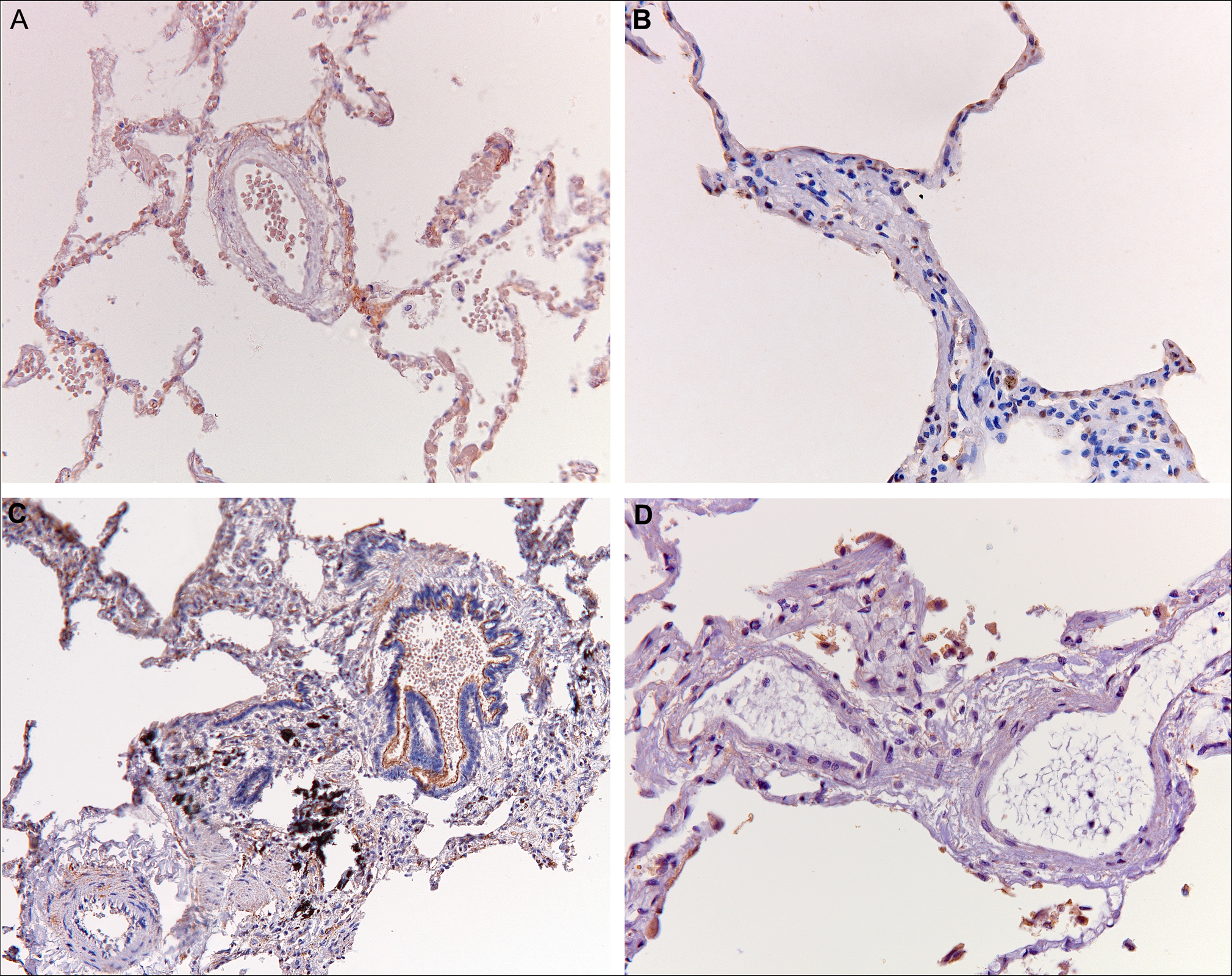

Supplement: Additional file 3 — Isoptype-matched control staining. additional file with representative figures of isoptype-matched control stainings of SScPAH-, IPAH- and PVOD-staining [file ar3315-S3.TIFF]
